# Supplementary material for: Reliable Discrimination of Green Coffee Beans Species: A Comparison of UV-Vis-Based Determination of Caffeine and Chlorogenic Acid with Non-Targeted Near-Infrared Spectroscopy
Source: Foods. 2020 Jun 16;9(6):788. doi: 10.3390/foods9060788 (PMC7353486; doi:10.3390/foods9060788)
Supplement: Supplementary file 1 [file foods-09-00788-s001.pdf]

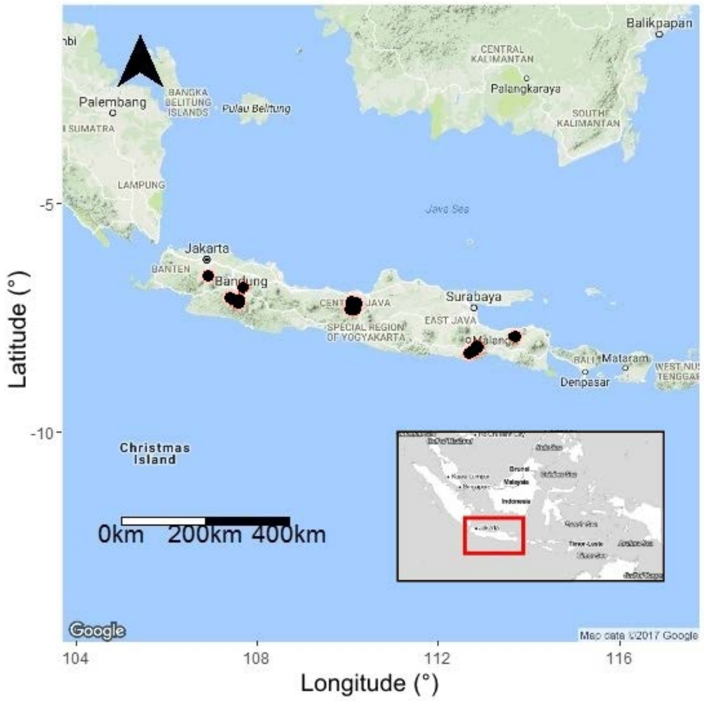

Figure S1. Map of sampling area in Java island, Indonesia. Black dots represent green bean sampling locations which were plotted using R application packages ggmap and ggsn.

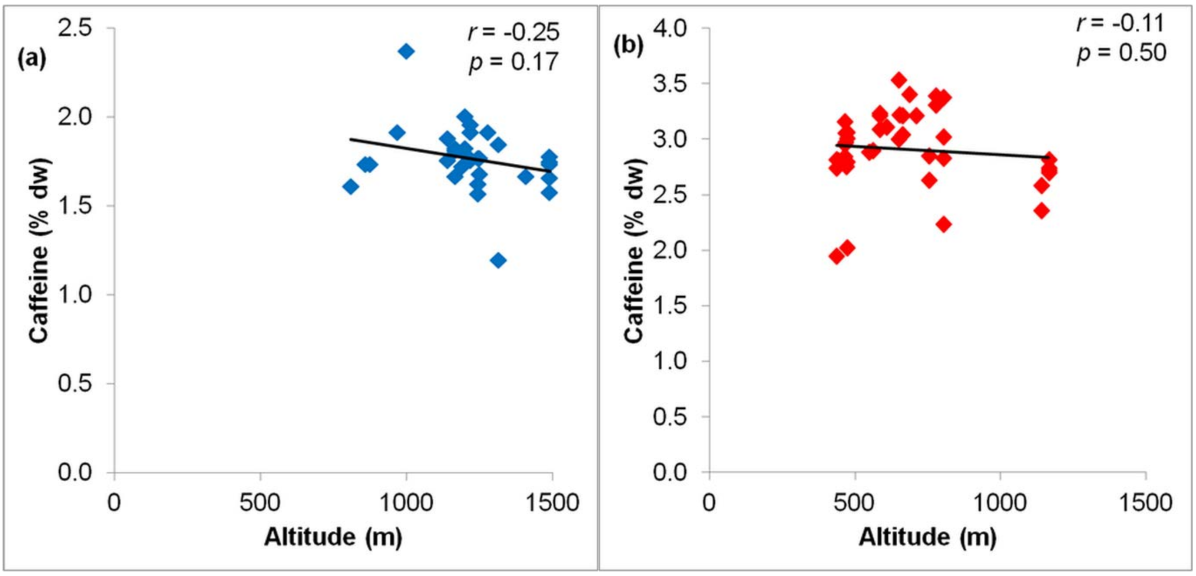

Figure S2. The correlation between caffeine content and altitude. (a) Arabica. (b) Robusta.

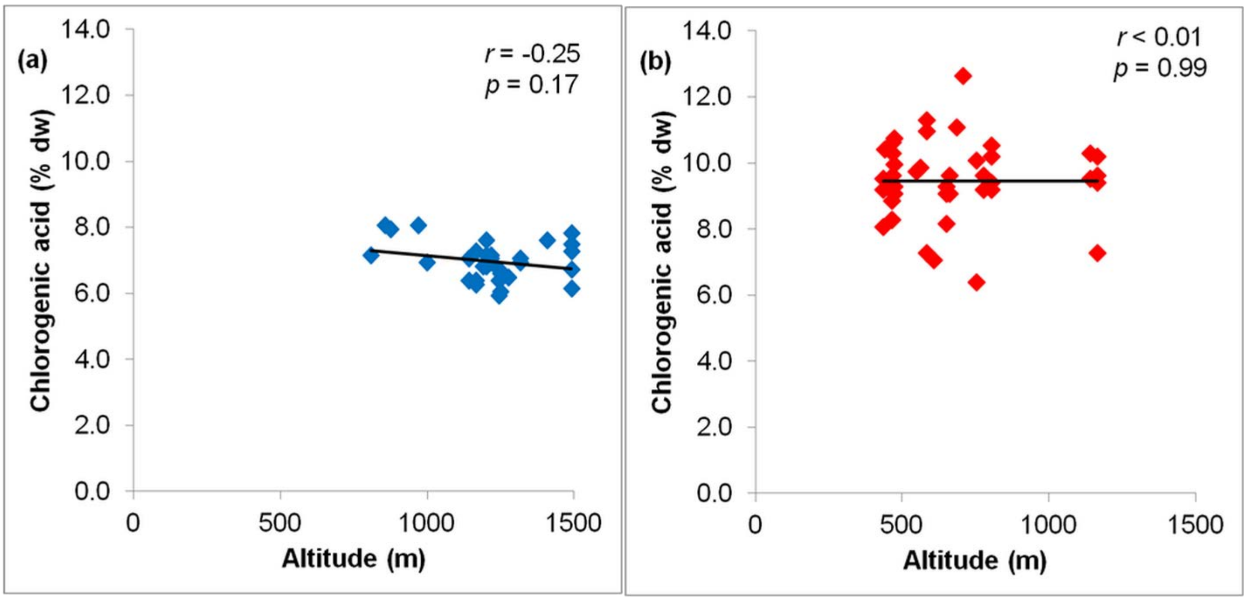

Figure S3. The correlation between chlorogenic acid content and altitude. (a) Arabica. (b) Robusta.

Table S1. Equations

No	Parameter	Equation
1	Beer-Lambert	$A = \mathcal{E} * c * l$<br><br>Where:<br>A = absorbance<br>ε = the molar decadic absorption coefficient,<br>c = the concentration of the absorbing compound.<br>l = distance in the absorbing medium
2	Caffeine content (% dw)	$\frac{\mathrm{C} * \mathrm{V}}{\mathrm{cf} * \mathrm{S}} * 100\%$<br><br>Where:<br>C = measured concentration (ppm)<br>V = sample volume (ml)<br>S = dry weight of sample (mg)<br>cf = conversion factor (0.001 mg ml^{-1} ppm^{-1})
3	Chlorogenic content (% dw)	$\frac{\mathrm{C} * \mathrm{V}}{\mathrm{cf} * \mathrm{S}} * 100\%$<br><br>Where:<br>C = measured concentration (ppm)<br>V = sample volume (ml)<br>S = dry weight of sample (mg)<br>cf = conversion factor (0.001 mg ml^{-1} ppm^{-1})

Table S1. Continue

No	Parameter	Equation
4	Root mean squared error of prediction (RMSEP)	$\sqrt{\frac{1}{N}\sum(\tilde{y}_i - y_{i,ref})^2}$ <br>where *N*= the size of the test set $\tilde{y}_i$ and $y_{i,ref}$ = the prediction and reference value for sample *i*, respectively

Table S2. Statistical parameters of several pre-processing method on diffuse reflectance (log 1/R) spectra by partial least squares discriminant analysis (PLS-DA).

No	Pre-processing method	LV	R^2 calibration	RMSEC	R^2 prediction	RMSEP
1	Raw	7	0.8896	0.3266	0.7151	0.6005
2	Smoothing<br>Moving average 3 segments	7	0.8896	0.3266	0.8893	0.327
3	Smoothing<br>Moving average 7 segments	7	0.8896	0.3265	0.7148	0.6162
4	Smoothing<br>Moving average 9 segments	7	0.8897	0.3265	0.7146	0.6276
5	Smoothing<br>Moving average 11 segments	7	0.8896	0.3266	0.7144	0.6426
6	Smoothing<br>Moving average 15 segments	7	0.8895	0.3268	0.7137	0.6837
7	Smoothing<br>Moving average 19 segments	7	0.8891	0.3273	0.7127	0.7375
8	Smoothing<br>GaussianFilter 3 segments	7	0.8895	0.3268	0.7148	0.6014
9	Smoothing<br>GaussianFilter 7 segments	7	0.8891	0.3273	0.7136	0.609
10	Smoothing<br>GaussianFilter 11 segments	7	0.8889	0.3277	0.7121	0.623
11	Smoothing<br>GaussianFilter 15 segments	7	0.8886	0.3281	0.7101	0.6441

Table S2. Continue

No	Pre-processing method	LV	R^2 calibration	RMSEC	R^2 prediction	RMSEP
12	Smoothing<br>GaussianFilter 19 segments	7	0.8882	0.3286	0.7077	0.6729
13	SavitskyGolay<br>1st derivative, 2 Polinomial order	4	0.9727	0.8829	0.0129	1.0143
14	SavitskyGolay<br>2st derivative, 2 Polinomial order	2	0.85916	0.3689	0.0687	1.557
15	SavitskyGolay<br>3st derivative, 3 Polinomial order	2	0.8371	0.3967	0.1772	2.003
16	OSC	6	0.8877	0.3295	0.5615	8.305
17	MSC	3	0.8526	0.3774	0.8134	0.4734
18	EMSC	6	0.9139	0.2884	0.9049	0.3641
19	Normalization<br>area	7	0.9317	0.257	0.903	0.3745
20	Normalization<br>mean	6	0.9317	0.257	0.903	0.3745
21	Baseline<br>Baseline Offset	6	0.9422	0.2364	0.7071	0.5933

Table S2. Continue

No	Pre-processing method	LV	R^2 calibration	RMSEC	R^2 prediction	RMSEP
22	Baseline<br>Linear Baseline Correction	6	0.8977	0.3144	0.5519	0.778
23	Baseline<br>Baseline offset +Linear Baseline Correction	7	0.894	0.32	0.7152	0.6338

LV: latent variables, R^2: the coefficient of determination, RMSEC: root mean square error of calibration, RMSECV: root mean square error of cross validation, RMSEP: root mean square error of prediction.

Table S3. Caffeine and chlorogenic acid content in different species and origin of green coffee beans samples.

Location	Origin	Species	Variety	Altitude (m)	Longitude	Latitude	Caffeine (% dw)	Chlorogenic acid (% dw)
1	West Java	Robusta	Wild	548	106.92	-6.59	2.88	9.73
2	West Java	Arabica	Java Preanger	1243	107.68	-6.82	1.62	5.93
	West Java	Arabica	Tim-tim	1243	107.68	-6.82	1.57	6.37
	West Java	Arabica	Linie S 795	1243	107.68	-6.82	1.77	6.71
3	West Java	Arabica	Sigararutang	1140	107.5	-7.11	2.36	10.29
	West Java	Robusta	Wild	1140	107.5	-7.11	2.58	9.50
	West Java	Robusta	Wild	1140	107.5	-7.11	1.88	6.37
	West Java	Arabica	Sigararutang	1140	107.5	-7.11	1.76	7.04
4	West Java	Arabica	Sigararutang + Linie S 795	1315	107.4	-7.06	1.20	7.04
	West Java	Arabica	Sigararutang + Linie S 795	1315	107.4	-7.06	1.85	6.93
5	West Java	Arabica	Tim-tim	1490	107.59	-7.17	1.58	7.27
	West Java	Arabica	Sigararutang	1490	107.59	-7.17	1.74	6.71
	West Java	Arabica	Linie S 795+Sigararutang+Andungsari	1490	107.59	-7.17	1.78	7.49
	West Java	Arabica	Sigararutang	1490	107.59	-7.17	1.65	6.15
	West Java	Arabica	Sigararutang	1490	107.59	-7.17	1.73	7.83
6	West Java	Arabica	Linie S 795+Sigararutang+Andungsari+Ateng	1409	107.56	-7.18	1.67	7.60
7	West Java	Arabica	Linie S 795+Ateng+Tim-tim+Preanger	1165	107.56	-7.11	2.72	10.18
	West Java	Arabica	Linie S 795+Ateng+Tim-tim+Preanger	1165	107.56	-7.11	2.82	9.62
	West Java	Robusta	Wild	1165	107.56	-7.11	2.69	7.27
	West Java	Robusta	Wild	1165	107.56	-7.11	2.74	9.39
	West Java	Robusta	Wild	1165	107.56	-7.11	1.67	7.27
	West Java	Robusta	Wild	1165	107.56	-7.11	1.81	6.37
	West Java	Arabica	Wild	1165	107.56	-7.11	1.82	6.26

Table S3. Continue

Location	Origin	Species	Variety	Altitude (m)	Longitude	Latitude	Caffeine (% dw)	Chlorogenic acid (% dw)
8	West Java	Arabica	Na	1217	107.69	-6.85	1.91	7.04
	West Java	Arabica	Na	1217	107.69	-6.85	1.76	7.16
	West Java	Arabica	Na	1217	107.69	-6.85	1.96	6.93
9	Central Java	Arabica	Na	1200	Na	Na	3.04	9.06
	Central Java	Arabica	Na	1200	Na	Na	3.21	9.62
10	Central Java	Robusta	Na	663	110.07	-7.18	1.82	7.16
	Central Java	Robusta	Na	663	110.07	-7.18	2.00	7.60
11	Central Java	Robusta	BP409	650	110.09	-7.14	3.00	9.28
	Central Java	Robusta	BP534	650	110.09	-7.14	3.53	8.16
12	Central Java	Robusta	BP534	804	110.07	-7.19	3.38	10.51
	Central Java	Robusta	BP409	804	110.07	-7.19	2.24	9.17
13	Central Java	Robusta	BP534	585	110.13	-7.18	3.21	7.27
	Central Java	Robusta	Wild	585	110.13	-7.18	3.23	11.29
	Central Java	Robusta	BP42	585	110.13	-7.18	3.09	10.96
14	Central Java	Robusta	Wild	609	110.16	-7.18	3.11	7.04
15	Central Java	Robusta	BP42	687	110.18	-7.18	3.40	11.07
16	Central Java	Robusta	BP534	652	110.17	-7.33	3.22	9.06
	Central Java	Arabica	Linie S 795	Na	Na	Na	2.23	8.83
17	Central Java	Robusta	Wild	754	110.12	-7.22	2.85	10.06
	Central Java	Robusta	BP42+BP534	754	110.12	-7.22	2.63	6.37
18	Central Java	Robusta	BP42	804	110.08	-7.21	2.83	10.18
	Central Java	Robusta	BGN371	804	110.08	-7.21	3.02	9.39
19	Central Java	Robusta	Wild	778	110.11	-7.21	3.31	9.62
	Central Java	Robusta	BGN371	778	110.11	-7.21	3.39	9.17

Table S3. Continue

Location	Origin	Species	Variety	Altitude (m)	Longitude	Latitude	Caffeine (% dw)	Chlorogenic acid (% dw)
20	Central Java	Robusta	BP534	709	110.13	-7.23	3.21	12.64
21	Central Java	Arabica	Kartika	1248	110.05	-7.33	1.77	6.04
	Central Java	Arabica	Linie S 795	1248	110.05	-7.33	1.68	6.60
22	East Java	Robusta	BP534	473	112.77	-8.22	3.01	10.73
	East Java	Robusta	BP534	473	112.77	-8.22	2.02	9.06
	East Java	Robusta	BP534	473	112.77	-8.22	3.06	9.95
	East Java	Robusta	BP534	473	112.77	-8.22	2.80	9.28
23	East Java	Robusta	Umbulsari+Wild	466	112.77	-8.22	2.84	8.27
	East Java	Robusta	Malamsari	466	112.77	-8.22	2.78	9.28
	East Java	Robusta	BP534	466	112.77	-8.22	3.15	8.27
24	East Java	Robusta	BP534	436	112.77	-8.23	2.82	9.50
	East Java	Robusta	Excelsa	436	112.77	-8.23	1.95	8.05
25	East Java	Robusta	BP534	441	112.77	-8.23	2.81	10.40
26	East Java	Robusta	BP534	436	112.77	-8.23	2.74	9.17
27	East Java	Robusta	Grembyong+BP534	469	112.77	-8.22	2.75	10.62
28	East Java	Robusta	Umbulsari	464	112.77	-8.22	2.95	8.83
29	East Java	Robusta	BP534	467	112.77	-8.22	2.97	9.62
	East Java	Robusta	BP534	467	112.77	-8.22	3.05	10.29
30	East Java	Arabica	Linie S 795	808	112.83	-8.17	1.61	7.16
31	East Java	Arabica	Linie S 795	874	112.83	-8.17	1.73	7.94
32	East Java	Arabica	Linie S 795	857	112.84	-8.17	1.73	8.05
33	East Java	Arabica	Linie S 795	1190	112.86	-8.14	1.72	6.82
34	East Java	Arabica	Linie S 795	1201	112.86	-8.14	1.79	6.82
35	East Java	Arabica	Na	968	112.84	-8.15	1.91	8.05

Table S3. Continue

Location	Origin	Species	Variety	Altitude (m)	Longitude	Latitude	Caffeine (% dw)	Chlorogenic acid (% dw)
36	East Java	Arabica	Na	999	112.84	-8.15	2.37	6.93
37	East Java	Arabica	Linie S 795	1277	113.69	-7.93	1.91	6.49
38	East Java	Robusta	BP 42+BP358	562	112.69	-8.28	2.90	9.84

+: there are several cultivated varieties in one plantation; Na: no available data; dw: dry weight.
